# The Locomotor Capabilities Index; validity and reliability of the Swedish version in adults with lower limb amputation

**DOI:** 10.1186/1477-7525-7-44

**Published:** 2009-05-23

**Authors:** Brita Larsson, Anton Johannesson, Ingemar H Andersson, Isam Atroshi

**Affiliations:** 1 Department of Rehabilitation Medicine, Hässleholm Hospital, SE-28125 Hässleholm, Sweden; 2 Department of Clinical Sciences, Lund University, Lund, Sweden, Ortopedteknik AB, Kristianstad Hospital, Kristianstad, Sweden; 3 Department of Health and Society, Kristianstad University, Kristianstad, Sweden; 4 Department of Clinical Sciences, Lund University, Lund, Sweden; 5 Department of Orthopedics, Hässleholm and Kristianstad Hospitals, Hässleholm, Sweden

## Abstract

**Background:**

The Locomotor Capabilities Index (LCI) is a validated measure of lower-limb amputees' ability to perform activities with prosthesis. We have developed the LCI Swedish version and evaluated its validity and reliability.

**Methods:**

Cross-cultural adaptation to Swedish included forward/backward translations and field testing. The Swedish LCI was then administered to 144 amputees (55 women), mean age 74 (40–93) years, attending post-rehabilitation prosthetic training. Construct validity was assessed by examining the relationship between the LCI and Timed "Up-and-Go" (TUG) test and between the LCI and EQ-5D health utility index in 2 subgroups of 40 and 20 amputees, respectively. Discriminative validity was assessed by comparing scores in different age groups and in unilateral and bilateral amputees. Test-retest reliability (1–2 weeks) was evaluated in 20 amputees (14 unilateral).

**Results:**

The Swedish LCI showed good construct convergent validity, with high correlation with the TUG (r = -0.75) and the EQ-5D (r = 0.84), and discriminative validity, with significantly worse mean scores for older than younger and for bilateral than unilateral amputees (p < 0.01), and high internal consistency (Cronbach alpha 0.95). In test-retest reliability the intraclass correlation coefficient was 0.91 (95% CI 0.79–0.96) but for the unilateral amputees was 0.83 (95% CI 0.56–0.94). Ceiling effect occurred in 23%.

**Conclusion:**

The Swedish version of the LCI demonstrated good validity and internal consistency in adult amputees. Test-retest reliability in a small subsample appears to be acceptable. The high ceiling effect of the LCI may imply that it would be most useful in assessing amputees with low to moderate functional abilities.

## Background

Patients with severe peripheral arterial disease or diabetes may require lower limb amputation and in Scandinavia these conditions account for more than 90% of all lower limb amputations [1]. The annual incidence of above-foot amputation ranges from 20 to 46 per 100,000 inhabitants [2,3]. In patients with lower limb amputation the primary aim of rehabilitation is to restore walking ability with prosthesis. Not all patients can receive prosthesis after amputation. The reported rate of prosthetic use following lower limb amputation related to peripheral arterial disease or diabetes has varied from 32% to 43% [4-6]. In addition, amputees successfully fitted with a prosthesis may differ in how much they use the prosthesis and in the type of activities they can perform with their prosthesis [7].

Walking ability with a prosthesis depends on several factors including patient's physical and mental status [8], the surgical method used [9], postoperative care, nutrition and pain relief [10] as well as the rehabilitation and prosthetic fitting procedures [6]. Lower limb amputation related to peripheral arterial disease or diabetes is usually performed on elderly patients who have multiple medical disorders, and the rehabilitation may be compromised by other illnesses such as stroke and heart failure or vascular problems involving the contralateral leg. An instrument that measures walking ability following amputation can therefore be used to trace changes in function related to comorbidity, treatment or rehabilitation.

The Locomotor Capabilities Index (LCI) is a 14-item questionnaire specifically designed to measure walking ability of lower-limb amputees. The LCI was developed in Canada in 1993 as part of the Prosthetic Profile of the Amputee questionnaire [11,12]. According to its developers the LCI "computes the global, basic, and advanced locomotor skills of the lower limb amputee with the prosthesis and assesses level of independence" [13]. The LCI has demonstrated good validity and reliability in adults with lower limb amputation and it has been found especially useful in daily clinical practices. It has been translated to several European languages and used in international studies [13]. Despite the relatively high incidence of above-foot amputations related to peripheral arterial disease or diabetes in Sweden [6], resulting in many prosthetic users, no valid and reliable measure of lower limb amputees' physical function with the prosthesis has been available in Swedish.

The purpose of this study was to perform a cross-cultural adaptation of the LCI to Swedish and evaluate the Swedish version for validity (convergent and discriminative) and reliability in lower limb amputees attending training after discharge from the hospital rehabilitation unit.

## Methods

### Procedure of translation

The procedure of cross-cultural adaptation of the English version of the LCI to Swedish was done in three steps [14]. First, the English version was translated to Swedish (forward translation) by 3 translators whose first language was Swedish, with one having no medical background. Based on consensus meeting a final version was created. In the second step, two bilingual persons whose first language was English independently re-translated the Swedish version into English (backward translation). Both were blinded to the concepts being investigated and one had no medical background. Finally, the translations were reviewed by a group consisting of 2 forward-translators, 1 backward-translator and one supervisor and discrepancies were resolved to achieve conceptual equivalence with the original version.

A pre-final version was created and tested on a reference group of 10 amputees attending training in a special after-rehabilitation training unit for amputees. The pre-final version performed well in the field-testing. However, the reference group suggested that a second version be created with lines between the questions for better readability as many amputees suffer from poor vision because of high age and/or diabetes. A final Swedish version of the LCI was then created (Additional file 1). The data from the field-testing were not used further in the analysis.

### Validation study

The Swedish version was assessed for validity (convergent and discriminative) and reliability (internal consistency and test-retest reliability) in a cross-sectional study conducted on a population of lower limb amputees attending training after discharge from the hospital rehabilitation unit with retest follow-up of a small subsample of the participants.

### Participants

Participants from our rehabilitation unit (Hässleholm-Kristianstad Hospitals) as well as from three other rehabilitation units in Sweden (one in Gothenburg, and two in Stockholm) were recruited for this study. The aim of these training units that are usually located in larger hospitals in Sweden is to help amputees who had undergone rehabilitation with prosthesis to maintain their mobility level. The training program is offered to amputees after the conclusion of routine prosthetic rehabilitation and participation is voluntary.

**Figure 1:**
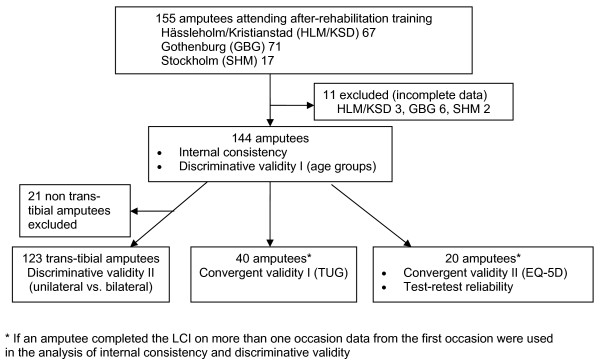
**Flow diagram of the participants in the validity and reliability analyses**.

The inclusion criteria for this study were age 40 years or older, lower limb amputation up to trans-femoral level, and that the amputee was fitted with a prosthesis. Data from all four rehabilitation units included gender, age, and amputation level, and data for the amputees from Hässleholm-Kristianstad also included date of amputation and of receiving the prosthesis. One hundred and fifty five amputees fulfilled the inclusion criteria (67 from Hässleholm-Kristianstad, 71 from Gothenburg, and 17 from Stockholm), of whom 11 were excluded because of incomplete data (Figure 1). The participants were representative of the amputee population in Sweden with the most common cause of amputation being peripheral arterial disease with or without diabetes and less commonly infection or fracture [6].

The data were collected from September 2003 through December 2007. The study population consisted of 144 amputees; 55 women, mean age 75 (range 40–93) years, and 89 men, mean age 73 (range 44–91) years (Table 1).

**Table 1:** Characteristic of the study population

	Discriminative validity I & Internal consistency	Discriminative validity II	Convergent validity I	Convergent validity II & Test-retest reliability
Number of amputees	144	123	40	20
Age, mean (range) yrs	74 (40–93)	74 (40–93)	74 (41–89)	76 (41–91)
Women, n (%)	55 (38)	50 (41)	15 (38)	10 (50)
Unilateral amputees, n (%)				
TT	110 (76)	110 (89)	40 (100)	13 (65)
TF/KD	18 (13)	0	0	1 (5)
Bilateral amputees, n (%)				
TT + TT/AD	14 (9.7)	13 (11)		5 (25)
TT + TF/KD	2 (1.3)	0		1 (5)
Time from prosthetic fitting to LCI testing, mean (range) wks	38 (3–418)^†^	38 (3–418)^†^	20 (3–135)	91 (4–231)

^†^Data for 64 amputees from Hässleholm-Kristianstad (not available for amputees from the other 3 centers)

TF, trans-femoral; KD, knee disarticulation; TT, trans-tibial; AD, ankle disarticulation

All participants from Hässleholm/Kristianstad were informed of the aim of the study and gave their written consent. Data from the other rehabilitation units contained no personal identifying information. The study was approved by the Local Ethics Committee.

### Questionnaires and mobility test

#### Locomotor Capabilities Index

The LCI consists of 14 items that measure one general construct, the locomotor capabilities with the prosthesis. Two subscales emerge from this general construct; basic abilities (7 items) and advanced abilities (7 items). The items inquire about the ability to perform activities and the level of independence while performing these activities. Each of the 14 items is graded on a 4-point ordinal scale; 0 (not able to), 1 (yes, with help from other person), 2 (yes, with supervision) and 3 (yes, independently). The total LCI score is the sum of the item scores and can range from 0 (worst) to 42 (best). Similarly, subscale scores for basic and advanced capabilities with the prosthesis can range from 0 to 21. The LCI is intended for self-administration but can also be administered in a face-to-face or telephone interview. The time needed to complete the LCI is approximately five minutes [11,12].

#### EQ-5D

The EQ-5D is a measure of health-related quality of life composed of 5 items covering 5 dimensions (mobility, self-care, usual activities, pain/discomfort, and anxiety/depression). Each item has 3 response levels: 1 (no problem), 2 (some problems), and 3 (unable to do for the first 3 items, or severe problems for the last 2 items). The preference weights for the EQ-5D index have been generated previously in the United Kingdom from a random general population sample using the time trade-off method of health evaluation [15]. The EQ-5D index ranges from 1.0 (no problem with any of the 5 dimensions), to -0.594 (extreme problems with all 5 dimensions). In this study the Swedish version of the EQ-5D was used. The EQ-5D is widely used, has shown to be reliable and valid in the Swedish general population, and is easy to complete [16].

#### Timed "Up-and-Go" Test

In the Timed "Up-and-Go" (TUG) test the participant is asked to, as fast as possible, rise from a chair, walk three meters with his/her ordinary walking aid, turn around, walk back and sit down again in the chair and the result is measured in seconds. The TUG test is easy to use in clinical settings and it has been shown to be valid and reliable in testing of function in an elderly population [17].

#### Evaluation of validity

We examined the completeness of item responses, the distribution of the scores, and the extent of ceiling and floor effects in the results from all 144 participants. We assessed construct validity of the LCI by testing a number of predefined hypotheses regarding its relationship with other measures of function and health (convergent validity) and its ability to discriminate among groups expected to differ in locomotor capabilities (discriminative validity) [18]. The number of participants included in the different analyses is shown in Figure 1.

Convergent validity [19] was determined by comparing the LCI results with the TUG test and EQ-5D results in 2 subgroups of amputees. We hypothesized that better LCI scores would have moderate or strong correlation (> 0.5) [20] with better TUG values, and that the LCI would correlate at least moderately with the EQ-5D index in a positive direction (i.e., better function with the prosthesis would correlate with better EQ-5D index). The correlation between the LCI scores and the TUG test results and the EQ-5D scores were calculated with the Spearman correlation coefficient (r). A correlation coefficient of at least 0.7 has been proposed as a standard for correlation in validity studies [21].

Discriminative validity was evaluated by comparing the LCI scores among amputees in different age groups and in unilateral and bilateral amputees. We hypothesized that younger amputees would have better LCI scores than older amputees and that unilateral trans-tibial amputees would have better scores than bilateral trans-tibial amputees. We also analyzed the LCI with regard to whether the scores would differ significantly according to gender as one previous study reported better scores among men than among women [22]. For comparison of LCI scores among amputees in different age groups and in men and women data from all 144 amputees were analyzed with Kruskal-Wallis test and the Mann-Whitney test, respectively. For comparing LCI scores in unilateral and bilateral trans-tibial amputees, data from 123 amputees were analyzed with the Mann-Whitney test.

### Evaluation of reliability

#### Internal consistency

Internal consistency measures the homogeneity in a scale and the items should be at least moderately correlated with each other. Internal consistency was determined using Cronbach alpha coefficient and the 95% confidence intervals (CI) were calculated using the bootstrap method. Values between 0.70 and 0.95 have been proposed to indicate good internal consistency [21]. Internal consistency reliability of the LCI was assessed using the responses from all 144 participants.

#### Test-retest reliability

Test-retest reliability was evaluated in the same subgroup of 20 amputees that provided data for the validity analysis using the EQ-5D. The participants completed the LCI on two occasions with a mean interval of 11 (range 7–14) days. The test-retest LCI scores were analyzed with the intraclass correlation coefficient (ICC) using the two-way random and absolute agreement criteria. The ICC (1,1) and 95% CI were calculated for the total LCI as well as for the basic and advanced subscales for the unilateral and bilateral amputees. For the ICC (range 0 to 1) a value of 0.70 has been considered as acceptable reliability [19].

#### Ceiling and floor effects

Reliability and validity of an instrument may be influenced by the presence of high ceiling and/or floor effects. A ceiling or floor effect is considered present if more than 15% of the respondents achieved the highest or lowest possible score [21,23].

### Statistical analysis

The LCI scores (total, basic and advanced) were calculated and presented as means, medians and standard deviations. All statistical tests were 2-sided and a p-value of 0.05 was considered to indicate statistical significance. Data were analyzed with SPSS version 14.0 (SPSS Inc., Chicago, USA) and STATA 10.0 (StataCorp, College Station, Texas, USA).

## Results

### Score distribution

All the 144 participants answered all items. Basic item 1 "rising from a chair" and item 2 "walk indoors" had the highest mean scores (2.9 and 2.7 respectively) and the worst scores were registered for advanced items 5 and 6 "getting up and down a stair without a handrail", both scoring a mean of 1.2 (Table 2).

**Table 2:** The locomotor capabilities index (LCI) item scores (n = 144)

			%	%
Items*	Mean (SD)	Median	Highest score	Lowest score
Basic activities				
1. Get up from a chair	2.9 (0.5)	3	91	0.7
2. Walk indoors	2.7 (0.6)	3	82	1.4
3. Walk outside	2.3 (1.1)	3	63	13
4. Go up stairs, handrail	2.4 (0.9)	3	65	9.0
5. Go down stairs	2.4 (0.9)	3	65	9.0
6. Step up sidewalk curb	2.2 (1.1)	3	63	16
7. Step down sidewalk curb	2.2 (1.1)	3	63	16
LCI – Basic	17.1 (5.5)	21	54	0.7
Advanced activities				
1. Pick up object from floor	2.0 (1.3)	3	63	26
2. Get up from floor	1.6 (1.3)	1	42	26
3. Walk outside	1.8 (1.3)	2	44	26
4. Outside in bad weather	1.6 (1.3)	2	42	35
5. Go up stairs without handrail	1.2 (1.2)	1	26	40
6. Go down stairs without handrail	1.2 (1.2)	1	26	40
7. Walk while carrying an object	1.7 (1.4)	3	51	38
LCI – Advanced	11.3 (7.8)	12	23	12
LCI – Total	28.5 (12.5)	33	23	0.7

*Each item has 4 score levels ranging from 0 (worst) to 3 (best); each LCI score (basic, advanced and total) is the sum of item scores

The mean total score was 28.5 (SD 12.5, median 33), the mean basic score was 17.1 (SD 5.5, median 21) and the mean advanced score 11.3 (SD 7.8, median 12).

### Convergent validity

In the subgroup that performed the TUG test, the mean LCI was 29.6 (range 2–42) and the mean TUG result was 34.2 (range 9–92) seconds. The correlation between the LCI and the TUG was strong (r = -0.75, 95% CI -0.89–0.56, p < 0.001). The mean EQ-5D index was 0.63 (SD 0.3; range -0.18–1.0). The correlation between the LCI and EQ-5D index was strong (r = 0.84, 95% CI 0.58–0.95, p < 0.001).

### Discriminative validity

**Figure 2:**
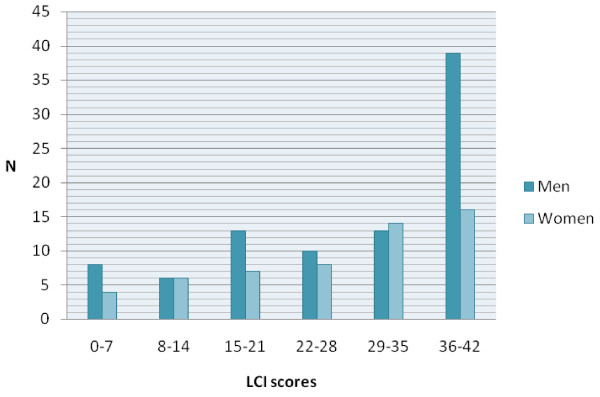
**Distribution of the LCI scores (N = 144)**.

The mean LCI score for the amputees in younger age groups was significantly better than that for amputees in older age groups (Table 3). The mean LCI score for unilateral amputees was 32.5 (SD 9.7) and for bilateral amputees was 14.9 (SD 12.5) (p < 0.001). The mean total score for women was 27.2 (SD 11.8, median 30) and for men 29.2 (SD 12.9, median 33), the difference was not statistically significant (p = 0.2), but LCI scores of 36 or higher were more common among men than women (39 men compared to 16 women) (Figure 2).

**Table 3:** The locomotor capabilities index (LCI) in different age groups (n = 144)

Age group yr	Number of amputees n (%)	LCI mean (SD)*
40–59	11 (7.6)	39 (4)
60–69	29 (20.1)	32 (12)
70–79	54 (37.5)	27 (12)
80+	50 (34.7)	25 (13)

* p = 0.001

### Internal consistency

Internal consistency was high; Cronbach alpha for the total LCI was 0.95 (95% CI 0.94–0.96), for basic activities was 0.93 (95% CI 0.91–0.95), and for advanced activities was 0.94 (95% CI 0.92–0.95). The item-total correlations ranged from 0.42 (item basic 1) to 0.85 (item advanced 3)

### Test-retest reliability

In the whole test-retest sample, the ICC for the total LCI was 0.91, for basic LCI was 0.88, and for advanced LCI was 0.92, and all 95% confidence intervals were above 0.70 (Table 4). The mean difference in the LCI scores between the two testing times was -1.6 for the total LCI and -0.8 for the basic and advanced LCI (all differences were statistically non-significant). Among the 14 unilateral amputees the ICC for the total LCI was 0.83, for the basic LCI was 0.66 and for the advanced was 0.92 (Table 4). For the 6 bilateral amputees the ICC for the total LCI was 0.90, for the basic 0.93 and for the advanced 0.59 and the mean score difference between the two testing times was -0.3, 0, and -0.3, respectively.

**Table 4:** Test-retest reliability

	Time 1	Time 2		
LCI	mean (SD)	mean (SD)	Mean difference (95% CI)	ICC (95% CI)
All amputees (n = 20)				
Total LCI	24.5 (13)	26.1 (14)	-1.6 (-4.3–1.1)	0.91 (0.79–0.96)
Basic	15.4 (7)	16.2 (7)	-0.8 (-2.4–0.8)	0.88 (0.72–0.95)
Advanced	9.2 (7)	10.0 (7)	-0.8 (-2.2–0.6)	0.92 (0.80–0.97)
Unilateral amputees (n = 14)				
Total LCI	29.9 (11)	32.1 (9)	-2.1 (-5.6–1.3)	0.83 (0.56–0.94)*
Basic	18.1 (5)	19.3 (4)	-1.1 (-3.3–1.0)	0.66 (0.24–0.87)*
Advanced	11.8 (7)	12.8 (7)	-1.0 (-2.5–0.5)	0.92 (0.78–0.97)

*The results are influenced by one outlier, for the other 13 amputees ICC basic is 0.78 (0.43–0.93) and total 0.89 (0.70–0.97).

ICC, intraclass correlation coefficient; CI, confidence interval

### Ceiling and floor effects

Of the 144 participants, 43 amputees had scores of 40 or higher and 33 (23%) had a maximum possible score (ceiling effect). High scores were more common among men than women. Only 1 amputee (0.7%) had a worst possible score and 12 had scores below 8 (Table 2).

## Discussion

This study shows that the Swedish version of the LCI has good validity. The predefined validity hypotheses were confirmed with good ability to discriminate among groups expected to differ in their locomotor capabilities and high correlations between the LCI and the TUG test and between the LCI and the EQ-5D. The reliability tests showed good internal consistency and the test-retest reliability in a small subsample was acceptable.

The measurement properties of the Swedish LCI are similar to those reported for the original English version. Assessment of validity of the original English version of the LCI [13] showed a significant correlation with the Functional Independence Measurement (FIM) test (Spearman correlation coefficient 0.62) and with the Rivermead Mobility Index (Spearman coefficient 0.75), which is similar to the correlation shown with the TUG test in our study, and assessment of reliability showed high internal consistency (Cronbach Alpha 0.95 for the total LCI and exceeding 0.90 for both subscales) and a high test-retest agreement (ICC = 0.80), which also is similar to the reliability results in our study. However, a very high Cronbach alpha may indicate possible item redundancy.

In our study, the mean LCI score was 28.5 in a population of 144 amputees with a mean age of 74 years. In a report from the developers of the LCI, a younger population (mean age 63 years) of 211 trans-tibial and 122 trans-femoral amputees had a mean LCI of 31.6 and 29.2, respectively [24]. Franchignoni et al. reported a mean LCI of 41 at the end of a rehabilitation program among 50 unilateral amputees with a median age of 51 years [25]. When comparing the LCI results in different studies the characteristics of the study populations should be taken into consideration. The LCI items that scored highest in our study were "getting up from a chair" and "walking indoors" and the lowest score was found for the item concerning climbing and descending a stair without a handrail, findings similar to those in previous studies [25,26].

The Swedish version could discriminate between unilateral and bilateral amputees and between younger and older amputees regarding degree of independence in performing locomotor activities. These findings support other studies that have demonstrated the usefulness of LCI in detecting differences in mobility [24].

In our study men had ceiling LCI scores more often than women but there were no statistically significant differences in the mean scores. In a study that analyzed predictors of good function after major lower limb amputation, Hermodsson et al. found male sex to be a statistically significant predictor and that men were three times more likely than women to achieve good function [22].

In our study a high correlation of -0.75 between LCI and TUG was found. A correlation of -0.64 was reported by Miller et al. who studied 55 amputees [27]. The TUG test is an objective test compared to the subjective nature of the LCI. The TUG test also shows how the patient's safety thinking works in a stressed situation; for example, whether they take the time to lock the wheels on the walking frame. Falls are common among amputees and one cause of decreasing function is fall injuries [28].

Our results showed a strong correlation between the LCI and self-perceived health measured with the EQ-5D. Walking is a fundamental human ability and seems to be strongly correlated to health. Using the Nottingham Health Profile, Pell et al. found that amputees with vascular disease reported significantly greater problems with mobility, social isolation, lethargy, pain, sleep and emotional disturbance than controls, but mobility was the only significant independent factor in a regression analysis [29]. The authors stated "the overall quality of life of amputees is likely to be enhanced by concentrating rehabilitation efforts on improving mobility". We believe that wheelchair mobility should be part of the rehabilitation program for amputees and we consider amputees to be "functional prosthetic users" even if they use a wheelchair for most of the day but are provided with prosthesis and are independent in transfers and can walk a few steps.

Despite the strong correlation with the LCI, the EQ-5D is a measure of health-related quality of life and does not specifically measure an amputee's function with the prosthesis. In addition, the ability of the EQ-5D to detect change in amputees' function over time has not been evaluated. In a study that used the EQ-5D in amputees with diabetes and foot ulcers in our region (including patients similar to the amputees in our study), the authors found that patients who had undergone major amputation had worse EQ-5D index than patients who achieved primary healing and those who had undergone minor amputation. The 26 amputees in that study had a mean EQ-5D index of 0.31 compared with 0.63 found among the amputees in our study [30]. One possible explanation is that the amputees in our study were participating in an after-rehabilitation training program.

The test-retest reliability of the total LCI, measured with the ICC in the whole test-retest sample, was comparable to that previously reported in other studies. Miller et al. [27] compared the LCI with two other self-report scales among 55 unilateral amputees (72% below-knee and 28% above knee), and reported that the ICC for the LCI was 0.88, for the Hougton scale was 0.85, and for the Prosthetic Evaluation Questionnaire was 0.77. One limitation in our study is the small test-retest sample size of 20 amputees in the evaluation of test-retest reliability. Recently, a research group stated that "no criteria have been defined for the required sample size of studies assessing measurement properties" and considered "a sample size of at least 50 patients adequate for the assessment of the agreement parameter, based on a general guideline by Altman [31]" and an ICC of 0.70 as minimum standard for reliability [21].

Different standards for acceptable ICC values have been proposed and a common recommendation is that measures intended for clinical use should have ICC exceeding 0.90 whereas for research purposes ICC of 0.70 has been considered acceptable [19]. Although the ICC values for the whole test-retest sample in our study were close to 0.90, the 95% confidence intervals were lower but still above 0.70 even for the two subscales. The inclusion of bilateral amputees in the test-retest sample may be considered problematic because it may increase the variability of the reliability coefficient and therefore may inflate the reliability [32]. In the subsample of unilateral amputees the ICC values were lower particularly for the basic LCI. Although the ICC values for the unilateral amputees were near or above levels considered acceptable, they were based on a small sample size and subsequently had wide confidence intervals. A study with a larger sample of unilateral trans-tibial amputees would be needed to further assess test-retest reliability and to confirm that the test-retest reliability is adequate for clinical use.

Ceiling effects with the use of LCI have been reported previously; one study reported a best possible score in 46% of 50 amputees (mean age 51 years) [25], and another in 40% of 329 amputees (mean age 60 years) [27]. In our study, the same pattern was observed, despite the high age of the participants amputated because of peripheral arterial disease. To address the problem of the ceiling effect the LCI-5 has been designed, with item response 3 "yes, alone" changed to 3 "yes, alone with ambulation aids" and 4 "yes, alone without ambulation aids" [25]. The high ceiling effect may have contributed to the high value for internal consistency.

In clinical practice there is increasing need to evaluate the methods used in rehabilitation because of a greater emphasis on patient safety and a growing interest in health economics. Whatever the purpose of their use, the tests must show not only high reliability and validity but also be easy to use in a clinical setting. The LCI appears to meet those requirements with its ease of use in daily practice.

Amputees with a low level of function may not know whether or not they can perform the activities inquired about in the questionnaire. Elderly amputees may, for safety reasons, have stopped performing certain activities when they are alone, and some may always use their wheelchair when outdoors. We have sometimes found it valuable that the amputees are given the opportunity to try to perform some of the activities in the presence of the test administrator. For instance, for the item "getting up after falling" the amputee could be allowed to sit on a mattress on the floor and try to rise up and stand with the help of a chair and "walking while carrying an object" could be exemplified as walking 10 meters while carrying a glass of water. Due to the highly functional nature of the items in the LCI, these tests may be useful, for instance when rehabilitation goals are defined together with the amputees [13]. However, care should be taken not to change the focus of the test from being a self-administered test to an observed test. Franchignoni et al. suggested guidelines for item scoring (e.g. carrying an object) as a possible improvement of the LCI [25].

The methods used for lower limb amputation and rehabilitation following amputation may differ between units, even when treating similar patients, and the differences may involve chosen levels of amputation, edema treatment, and time chosen for prosthetic fitting [2,6,22]. Finding the best practice would require a standardized protocol for measuring rehabilitation progress after lower limb amputations. The LCI could be a useful tool in this context.

## Conclusion

The Swedish version of the LCI demonstrated good validity and internal consistency in adult amputees. Test-retest reliability in a small subsample appears to be acceptable. The ceiling effect was high, which may imply that it would be most useful in assessing amputees with low to moderate functional abilities.

## Competing interests

The authors declare that they have no competing interests.

## Authors' contributions

BL, HIA, AJ and IA conceived of and designed the study. BL, AJ and IA analyzed and interpreted the data. AJ, BL and IA performed the statistical analysis. BL and AJ drafted the paper and IA critically revised it for important intellectual content. All authors read and gave approval of the final manuscript.

## Supplementary Material

Additional file 1 **Swedish version of LCI**. The translated version of the Locomotor Capabilities Index Click here for file
