# Supplementary material for: The Locomotor Capabilities Index; validity and reliability of the Swedish version in adults with lower limb amputation
Source: Health Qual Life Outcomes. 2009 May 23;7:44. doi: 10.1186/1477-7525-7-44 (PMC2696440; doi:10.1186/1477-7525-7-44)
Supplement: Additional file 1 — Swedish version of LCI. The translated version of the Locomotor Capabilities Index [file 1477-7525-7-44-S1.doc]

# LOCOMOTOR CAPABILITIES INDEX

# Funktionsindex för benamputerade

# Namn……………………………………

Personnummer……………………………

Amputationsnivå ………..………………

Datum……………………………………

Även om Du inte använder Din protes just nu, hur uppfattar Du Din förmåga att utföra

följande aktiviteter med protesen på?

**(Personlig intervju)**

Skala:

| 0 = nej | 1 = ja, med hjälp av annan person | 2 = ja, med tillsyn | 3 = ja, självständigt |
| --- | --- | --- | --- |

Ringa in en siffra för varje påstående.

| GRUNDLÄGGANDE AKTIVITETER | SKALA | | | |
| --- | --- | --- | --- | --- |
| 1. Resa Dig från en stol | 0 | 1 | 2 | 3 |
| 1. Gå inomhus | 0 | 1 | 2 | 3 |
| 1. Gå utomhus på jämn mark | 0 | 1 | 2 | 3 |
| 1. Gå uppför en trappa med hjälp av ledstång | 0 | 1 | 2 | 3 |
| 1. Gå nerför en trappa med hjälp av ledstång | 0 | 1 | 2 | 3 |
| 1. Kliva uppför en trottoarkant | 0 | 1 | 2 | 3 |
| 1. Kliva nerför en trottoarkant | 0 | 1 | 2 | 3 |
| Poängsumma | **/21** | | | |
| KRÄVANDE AKTIVITETER |  | | | |
| 1. Plocka upp ett föremål från golvet (när Du står med Din protes på) | 0 | 1 | 2 | 3 |
| 1. Resa Dig upp från golvet (t ex om Du fallit) | 0 | 1 | 2 | 3 |
| 1. Gå utomhus på ojämn mark (t ex gräs, grus eller i sluttning) | 0 | 1 | 2 | 3 |
| 1. Gå utomhus i dåligt väder (t ex snö, regn eller halka) | 0 | 1 | 2 | 3 |
| 1. Gå uppför några få trappsteg utan hjälp av ledstång | 0 | 1 | 2 | 3 |
| 1. Gå nerför några få trappsteg utan hjälp av ledstång | 0 | 1 | 2 | 3 |
| 1. Gå och samtidigt bära ett föremål | 0 | 1 | 2 | 3 |
| Poängsumma | **/21** | | | |
| Total poängsumma | **/42** | | | |

© Gautier-Gagnon & Grisé, 19
